# Detecting Inter-Cusp and Inter-Tooth Wear Patterns in Rhinocerotids

**DOI:** 10.1371/journal.pone.0080921

**Published:** 2013-12-03

**Authors:** Lucy A. Taylor, Thomas M. Kaiser, Christoph Schwitzer, Dennis W. H. Müller, Daryl Codron, Marcus Clauss, Ellen Schulz

**Affiliations:** 1 Bristol Conservation and Science Foundation, c/o Bristol Zoo Gardens, Bristol, United Kingdom; 2 School of Biological Sciences, University of Bristol, Bristol, United Kingdom; 3 Biocenter Grindel and Zoological Museum, University of Hamburg, Hamburg, Germany; 4 Clinic for Zoo Animals, Exotic Pets and Wildlife, Vetsuisse Faculty, University of Zurich, Zurich, Switzerland; 5 National Park ‘Bavarian Forest’, Grafenau, Germany; 6 Florisbad Quaternary Research Department, National Museum, Bloemfontein, South Africa; Monash University, Australia

## Abstract

Extant rhinos are the largest extant herbivores exhibiting dietary specialisations for both browse and grass. However, the adaptive value of the wear-induced tooth morphology in rhinos has not been widely studied, and data on individual cusp and tooth positions have rarely been published. We evaluated upper cheek dentition of browsing *Diceros bicornis* and *Rhinoceros sondaicus*, mixed-feeding *R. unicornis* and grazing *Ceratotherium simum* using an extended mesowear method adapted for rhinos. We included single cusp scoring (EM(R)-S) to investigate inter-cusp and inter-tooth wear patterns. In accordance with previous reports, general mesowear patterns in *D. bicornis* and *R. sondaicus* were attrition-dominated and *C. simum* abrasion-dominated, reflecting their respective diets. Mesowear patterns for *R. unicornis* were more attrition-dominated than anticipated by the grass-dominated diet, which may indicate a low intake of environmental abrasives. EM(R)-S increased differentiation power compared to classical mesowear, with significant inter-cusp and inter-tooth differences detected. In *D. bicornis*, the anterior cusp was consistently more abrasion-dominated than the posterior. Wear differences in cusp position may relate to morphological adaptations to dietary regimes. Heterogeneous occlusal surfaces may facilitate the comminution of heterogeneous browse, whereas uniform, broad grinding surfaces may enhance the comminution of physically more homogeneous grass. A negative tooth wear gradient was found in *D. bicornis, R. sondaicus* and *R. unicornis*, with wear patterns becoming less abrasion-dominated from premolars to molars. No such gradients were evident in *C. simum* which displayed a uniform wear pattern. In browsers, premolars may be exposed to higher *relative* grit loads, which may result in the development of wear gradients. The second premolar may also have a role in food cropping. In grazers, high *absolute* amounts of ingested abrasives may override other signals, leading to a uniform wear pattern and dental function along the tooth row, which could relate to the observed evolution towards homodonty.

## Introduction

The Family Rhinocerotidae (rhinos) first appeared in the Late Eocene of Eurasia and was a remarkably successful and diverse mammalian group in the Neogene [1]. A small part of the former diversity remains in the extant rhinos, which are the largest remaining herbivores exhibiting dietary specialisations for both browse and grass [2]. Within this group, a broad spectrum of tooth morphology is exhibited. Browsing *Diceros bicornis* have lophodont cheek teeth, with a dominant ectoloph cutting edge, concave occlusal surface, uneven enamel thickness, low relative hypsodonty index, and a two-phase masticatory movement [3]. By contrast, grazing *Ceratotherium simum* have plagiolophodont teeth, with flattened occlusal surfaces, blunter tooth blades, uniform enamel thickness, high hypsodonty index and a unimodal, transverse masticatory movement [3]–[5]. The genetically determined tooth morphology is a result of the evolutionary history and adaptations of an animal to a specific dietary and/or habitat, whereas the tooth wear experienced throughout a lifetime represents a substantial proportion of an individual’s behavioural history [4], [6]. The adaptive value of tooth morphology in rhinos has not been widely studied [3], [7]. In particular, the wear-induced morphology seems to be strongly related to diet [6], [8], but individual cusp or tooth positions have not been considered.

Analyses of tooth wear using the ‘classical’ mesowear method developed by Fortelius and Solounias [6] have been used extensively in dietary and habitat reconstruction of ungulate species [6], [9]–[13]. Mesowear is based on the facet development of cheek tooth occlusal surfaces. The degree of facet development reflects the relative proportions of tooth-to-tooth contact (attrition) and food-to-tooth contact (abrasion) [6], [9], [14]. The entire surface of the tooth is affected by wear, but the mesowear method focuses on the buccal cutting edges of the enamel surface at the ectoloph [6], [9]. Mesowear treats tooth wear as two variables: occlusal relief (OR) and cusp shape (CS). Browsers’ teeth are characterised by high OR and sharp CS, which is interpreted as attrition-dominated wear, whereas grazers’ teeth are characterised by low OR and blunt CS, which is interpreted as abrasion-dominated wear [6]. Recently, an extended mesowear method (EM) was developed by Winkler and Kaiser [15] by introducing additional categories to produce a higher resolution for both, OR and CS. However, in ‘classical’ mesowear, different OR boundaries were used for rhinoceros (length/height = 0.03) compared to selenodonts and equids (0.1). Rhino teeth are asymmetrical, particularly in black rhinos where the anterior cusp is proportionally smaller than the posterior on the ectoloph [3]. Fortelius and Solounias [6] suggested not scoring CS in cusps altered by structural elements, such as the anterior cusp of rhinos. Therefore, in order to investigate the mesowear scores of rhinos at a higher resolution, the EM method needs to be further adjusted. A differential analysis of wear patterns along the tooth row will allow insights on the mechanics of ingestion and mastication and on the adaptive value of rhino tooth morphology. In addition, the diets of Pleistocene rhinos are debated, particularly *Stephanorhinus hemitoechus* [3], [8], [16]. An EM method could provide new insight into tooth wear patterns of both extant and extinct rhinos.

Here we report findings on the wear-induced tooth morphology in rhinos adapting the EM method of Winkler and Kaiser [15]. Extant rhinos are particularly suited for this study because they differ distinctively in their dietary traits, which is well-described in the literature (Table 1). The EM method [15] was further adapted for rhinos, including single cusp scoring for the anterior and posterior cusp (EM(R)-S), to test for wear characteristics between cusp and tooth positions along the tooth row. Mesowear analyses originally focused on the maxillary second molar [6], but have been extended to the maxillary and mandibular premolar 4 to molar 3 (P4-M3) in equids [10], [17] and maxillary M1-M2 in rhinos [8]. We expanded the analysis to the maxillary P2-M2.

**Table 1:** Grass consumption and habitats of free-ranging rhinoceros.

Species	Grass (%)	Habitat category	Habitat type	References
*Ceratotherium simum*	90–100	Open	Savannah	[47]–[50]
*Diceros bicornis*	0–9	Mixed	Savannah and bushveld	[50]–[57]
*Rhinoceros sondaicus*	0	Closed	Tropical rainforest	[58]–[60]
*Rhinoceros unicornis*	53–89*	Mixed	Riverine grasslands, also swamps and forests	[28]–[34]

Habitat category information from Mendoza and Palmqvist [36].

* 42–92% in a population 4–6 years after translocation Jnawali [30].

## Methods

### Material

Forty-eight museum specimens of wild black (*Diceros bicornis* Linnaeus, 1758, n = 22), greater one-horned (*Rhinoceros unicornis* Linnaeus, 1758, n = 11), white (*Ceratotherium simum* Burchell, 1817, n = 9) and Javan rhinoceros (*R. sondaicus* Desmarest, 1822, n = 6) from 15 zoological museums were investigated (Table S1). All museum specimens examined in this study are housed in publicly accessible collections and were examined and moulded with kind permission while visiting the respective museums. *Ceratotherium simum* was treated as one species, instead of dividing into *C. simum* and *C. cottoni* as suggested by Groves et al. [18], due to the small sample size. Only specimens with a known origin from the wild were used. Dental casts were produced of either the left or the right maxillary (upper) cheek tooth row of each specimen, depending on which side had been best preserved. Selected tooth rows were cleaned with ethanol or acetone, and a negative mould of each tooth row was made with Provil novo Light C.D. 2 fast set EN ISO 4823, type 3, light (Heraeus Kulzer GmbH, Hanau, Germany) and Provil novo Putty regular set EN 24823 (Heraeus Kulzer GmbH, Hanau, Germany) polysiloxane dental moulding putty. One-to-one positive casts of the dental moulds were subsequently produced by filling the moulds with epoxy resin Injektionsharz EP (Reckli-Chemiewerkstoff, Herne, Germany). The dental casts are stored at the Biocentre Grindel and Zoological Museum, University of Hamburg (ZMH), enabling continuous access to specimens.

**Figure 1:**
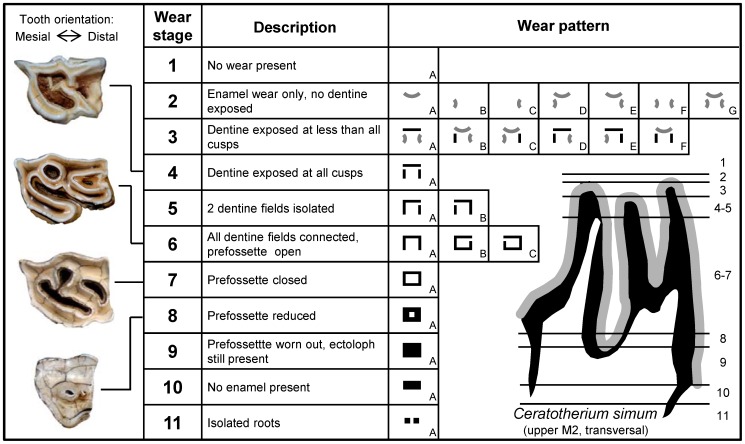
Dental wear stages of modern Rhinocerotidae. Upper left molars adopted from Kaiser et[20] and Payne [46]. Left = anterior, up = buccal. Grey sickle shaped symbols: only enamel wear, no dentin exposed. All other black areas symbolise exposed dentine fields. All dentine fields are surrounded by enamel crests. The scheme represents patterns frequently observed, early wear stages 2–5 are very variable and within a species several patterns may occur. Functional stages are considered as 6 in *Diceros bicornis* and *Rhinoceros sondaicus* and 6–7 in *R. unicornis* and *Ceratotherium simum*.

Maxillary tooth rows, including the permanent second, third and fourth premolar (P2, P3 and P4) and the first and second molar (M1 and M2), were analysed. Premolar 1 (P1) was excluded as P1 is not consistently present between all species and is often reduced [19]. Molar 3 (M3) was scored, but later excluded due to the reduced ectoloph of the M3 as compared to the other cheek teeth in *D. bicornis, R. unicornis* and *R. sondaicus*. As ontogeny can affect mesowear [6], [11] and the absolute age of the specimens was unknown, tooth wear stage was used to ensure all specimens were in the same dental functional stage and thereby excluding young and very old individuals. The wear stages chart of Kaiser et al. [20] was adapted for rhinos (Figure 1). The chart focuses on the prefossette in rhinos, as the postfossette did not appear to wear down consistently. In *D. bicornis* and *R. sondaicus*, stage 6 was considered the main functional stage and in *C. simum* stages 6–7. Stages 6–7 were also considered for *R. unicornis*, despite having a lower hypsodonty index than *D. bicornis* (1.59 compared to 2.24, respectively) [21], due to the flatter tooth shape. All broken cusps and pathological teeth were excluded from the analysis.

### Mesowear Development

The extended mesowear method of Winkler and Kaiser [15] (EM) includes four categories for OR of ‘high-high’ (height/length ≥0.25), ‘high’ (0.25–0.125), ‘high-low’ (<0.125–0.05) and ‘low’ (<0.05). While scoring we noted that the OR categories are difficult to estimate visually due to the asymmetry of rhino teeth, thus OR was measured separately in the anterior and posterior cusp position using digital callipers producing a single cusp score (EM-S). The height of the cusp was taken from the apex of the cusp (anterior or posterior) down to the line connecting the inner valley and the outside edge of the tooth, and the length was taken as the total length of the tooth. Total tooth length was used, rather than cusp length, because the divide between cusps was often ambiguous, whereas total tooth length was a consistent measure. No rhinos attained an OR score of ‘high-high’, indicating that the current boundary for ‘high-high’ is too high for rhinos. By contrast, 50% of browsing *D. bicornis* had a ‘high’ relief in the posterior cusp of the M2. Thus, we propose moving the OR ‘high-high’ (≥0.25) boundary for rhinos down to the ‘high’ (≥0.125) boundary as defined by Winkler and Kaiser [15].

**Figure 2:**
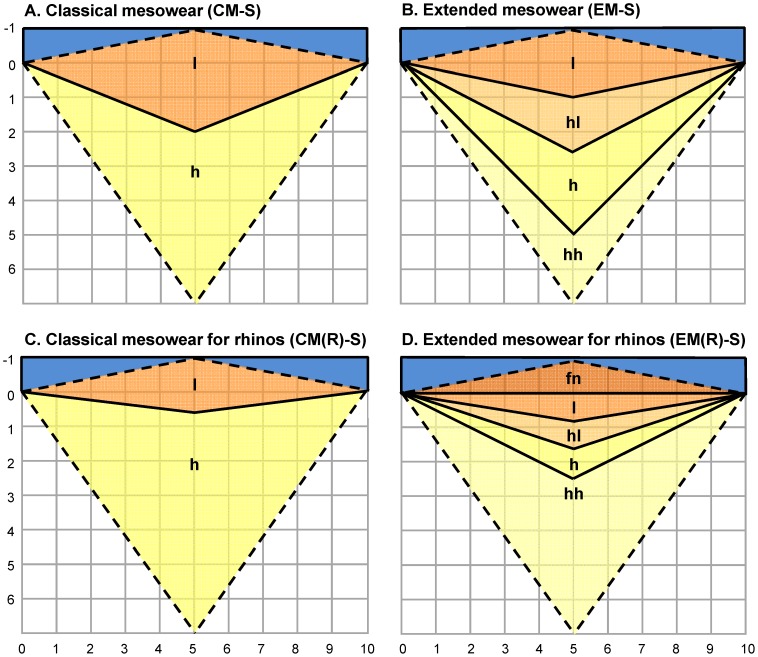
Visualisation of occlusal relief categories in classical and extended mesowear. Black solid lines indicate category boundaries. Dashed lines indicate potential relief patterns beyond these boundaries. Categories: fn = ‘flat-negative’, l = ‘low’, hl = ‘high-low’, h = ‘high’ and hh = ‘high-high’.

The classical mesowear method of Fortelius and Solounias [6] for rhinos (CM(R)) includes two categories of OR, ‘low’ and ‘high’, with a boundary of 0.03 for rhinos, which is substantially lower than the boundary used for selenodonts of 0.1. However, a boundary of 0.03 is visually and practically difficult to differentiate because the relationship between height and length is a recurring decimal of 1/33.33. Thus, instead of a visually difficult boundary we added another OR category of ‘flat-negative’ (fn; ≤0), denoting no or negative OR, to provide additional differentiation on a clear boundary. Between the new position of ‘high-high’ (≥0.125) and ‘flat-negative’, we then equally spaced the other categories of ‘high’ (<0.125–0.083), ‘high-low’ (<0.083–0.0417) and ‘low’ (<0.0417>0.00), transforming continuous tooth wear into simple ranked blocks (rank transformation; Figure 2). All of the new boundaries above the clear ‘flat-negative’-‘low’ boundary are on whole integers (‘high-high’ ≥1/8, ‘high’ ≥1/12 and ‘high-low’ ≥1/24), meaning visual differentiation is possible.

### Mesowear Scoring

**Figure 3:**
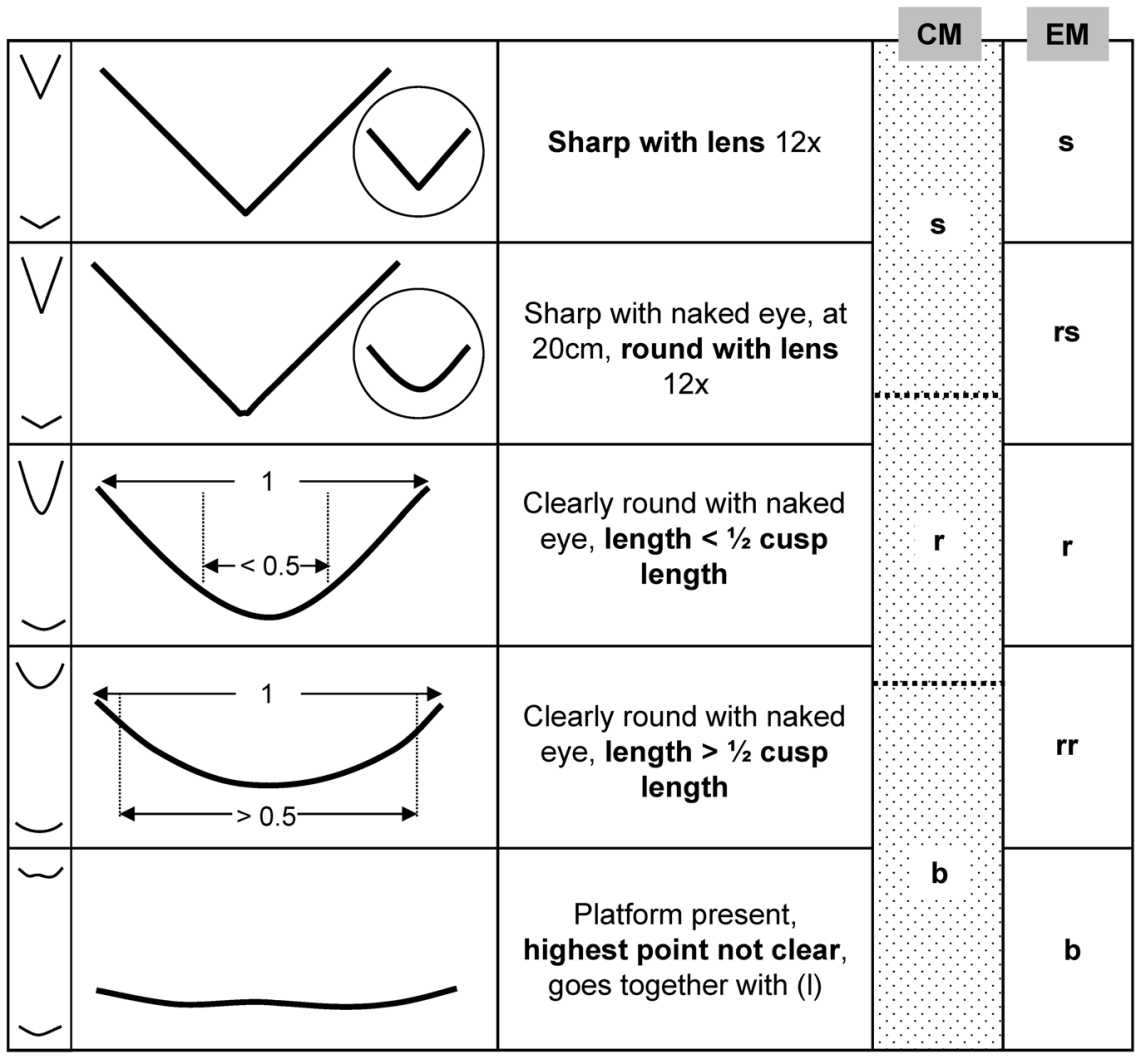
Cusp shape categories. Description of the cusp shape categories for the classical mesowear method of Fortelius and Solounias [6] (CM) and the enhanced mesowear method of Winkler and Kaiser [15] (EM). Both EM and EM adjusted for rhinos includes the same categories of cusp shape. Cusp shape categories: s = ‘sharp’, rs = ‘round-sharp’, r = ‘round’, rr = ‘round-round’ and b = ‘blunt’.

All specimens were then scored using single cusp scoring for the anterior and posterior cusp position separately using the classical mesowear method of Fortelius and Solounias [6] with rhino OR adjustment (CM(R)-S), the extended method of Winkler and Kaiser [15] (EM-S) and the method of Winkler and Kaiser [15] with OR adjustments for rhinos suggested in this study (EM(R)-S). CM(R)-S includes two categories of OR, high (height/length ≥0.03) and low (l; <0.03), and three categories of cusp shape, ‘sharp’, ‘round’ and ‘blunt’. EM-S includes four categories of OR, ‘high-high’ (height/length ≥0.25), ‘high’ (0.25–0.125), ‘high-low’ (<0.125–0.05) and ‘low’ (<0.05), and five categories of CS, ‘sharp’, ‘round-sharp’, ‘round’, ‘round-round’ and ‘blunt’. EM(R)-S includes five categories of OR, ‘high-high’ (≥0.125), ‘high’ (h; <0.125–0.083), ‘high-low’ (hl; <0.083–0.0417), ‘low’ (l; <0.0417>0.00) and ‘flat-negative’ (fn; ≤0), and the same CS scores as EM-S (Figure 3). A triplet hand lens (10x–18 mm) was used to score CS when required.

Each of these methods was then converted into a mesowear score for analysis. CM(R)-S results were converted into a combination score of 0 ‘high’ and ‘sharp’, 1 ‘high’ and ‘round’, 2 ‘low’ and ‘round’, 3 ‘low’ and ‘sharp’ and 4 ‘low’ and ‘blunt’ [22]. For EM-S and EM(R)-S, OR and CS were each converted into a score, which ranged from 0 ‘high-high’ up to 3 ‘low’ (EM-S) or 4 ‘flat-negative’ (EM(R)-S) in OR, and from 0 ‘sharp’ to 4 ‘blunt’ in CS. In order to compare CM(R)-S, EM-S and EM(R)-S, a mesowear score was calculated for EM-S and EM(R)-S by taking a mean of the OR and CS scores, with scores ranging from 0.0–3.5 (EM-S) or 0.0–4.0 (EM(R)-S; Table S2).

### Statistical Analyses

OR, CS and mesowear score mean values were calculated for all four species. Due to the small sample size of *R. sondaicus* (n = 4–6), inferential statistical analyses were not performed on this species. Mesowear analyses have thus far only been applied on the M1 and M2 for rhinos [8]; therefore we used these tooth positions to compare mesowear methods. Methods were compared by fitting linear mixed-effects (LME) models, with hierarchically nested fixed effects of species, cusp position (nested in species) and tooth position (nested in cusp position (nested in species)). Due to the broken cusp and wear stage exclusions, there were unequal sample sizes in each tooth and cusp position. In order to minimise the loss of data, balanced subsamples were taken separately for the anterior and posterior cusp within each tooth position. Totally balanced subsamples were tested, but resulted in a substantial loss of data without a significant difference to the results. Random subsamples were taken using the statistical program R version 2.15.2 [23]. Intraspecific differences between species were then tested using cusp position and tooth position (cusp position). Individual specimen was added as a random effect on all models. We used restricted maximum likelihood (REML), because it provides the most reliable estimates of the variance components [24], and calculated denominator degrees of freedom using Sattherthwaite’s approximation, due to the unbalanced design [25]. The same models and levels of nesting were used for all statistical tests to allow comparisons between methods and species.

Intraspecies differences within each cusp and tooth position along the whole tooth row for the CS score, OR score and mesowear score were then tested using LME models for each species using the EM(R)-S. Fixed effects were hierarchically nested as cusp position and tooth position (cusp position), and individual was added as a random effect. Cusp differences within each tooth position were subsequently tested with Welch Two Sample *t*-tests and tooth position differences were tested separately in the anterior and posterior cusp position using one-way analyses of variance (ANOVA) and Dunnett’s T3 post hoc test. Statistical analyses and data manipulation were conducted in IBM® SPSS® Statistics 19 software (SPSS Inc., Chicago, IL, USA) and in the open-source software R version 2.15.2 [23] using the packages doBy [26] and xlsx [27]. The significance level was set to *p*<0.05.

## Results

### Mesowear Development

In accordance with previous reports [6], [8], general mesowear patterns in *Diceros bicornis* and *Rhinoceros sondaicus* were attrition-dominated and *Ceratotherium simum* abrasion-dominated, reflecting their respective diets (Table 1). Mesowear patterns for *R. unicornis* were also attrition-dominated, in contrast to the abrasion-dominated wear signatures reported by Hernesniemi et al. [8] but similar to Fortelius and Solounias [6]. Tooth wear differences were detected in M1 and M2 between *D. bicornis, R. unicornis* and *C. simum* using all three mesowear methods when these species were analysed together (*p*<0.001). The extended method using single cusp scoring (EM-S and EM(R)-S) had higher intra-species resolution, with highly significant differences in both cusp and tooth positions between *D. bicornis, R. unicornis* and *C. simum* (LME: *p*<0.001) (Table 2).

**Table 2:** Comparison of mesowear methods using linear mixed–effects models.

			Mesowear method		
			CM(R)-S	EM-S	EM(R)-S
All species (3)	Species		**<0.001**	**<0.001**	**<0.001**
	Cusp (Species)		**0.004**	**<0.001**	**<0.001**
	Tooth (Cusp (Species))		0.173	**<0.001**	**<0.001**
*Diceros bicornis*	M1 (n = 21)	A	1.0±0.4	2.2±0.4	2.1±0.4
		P	0.5±0.5	1.7±0.3	1.4±0.5
	M2 (n = 22)	A	1.0±0.5	2.3±0.4	2.1±0.4
		P	0.5±0.5	1.5±0.4	1.0±0.4
	Cusp		**<0.001**	**<0.001**	**<0.001**
	Tooth (Cusp)		0.799	0.095	**0.004**
*Rhinoceros unicornis*	M1 (n = 11)	A	1.6±1.3	2.5±0.4	2.5±0.5
		P	1.4±1.0	2.4±0.6	2.3±0.6
	M2 (n = 11)	A	1.1±1.0	2.0±0.5	2.0±0.5
		P	0.8±0.4	1.7±0.5	1.3±0.6
	Cusp		0.281	**0.045**	**0.001**
	Tooth (Cusp)		0.109	**<0.001**	**<0.001**
*Ceratotherium simum*	M1 (n = 7)	A	3.7±0.8	3.4±0.2	3.4±0.3
		P	3.4±1.0	3.2±0.5	3.5±0.7
	M2 (n = 9)	A	3.7±1.0	3.3±0.5	3.4±0.5
		P	3.7±1.0	3.3±0.5	3.6±0.6
	Cusp		0.559	0.357	0.406
	Tooth (Cusp)		0.833	0.652	0.955

Mesowear score (mean values ±standard deviation) and *p*-values (significant values in bold) of hierarchically nested linear mixed-effects models using species, cusp and tooth position as fixed effects and individual as a random effect, for the classical mesowear method for rhinos of Fortelius and Solounias [6] (CM(R)-S), the extended mesowear method of Winkler and Kaiser [15] (EM-S) and the extended mesowear method adapted for rhinos (EM(R)-S). All methods use single cusp scoring.

In the M1 and M2 of *D. bicornis*, there were highly significant differences between cusp positions in all methods (*p*<0.001), but differences in tooth position were only detected by EM(R)-S (*F*_2,65.9_ = 5.95, *p* = 0.004). In the ‘classical’ mesowear method for rhinos (CM(R)-S), differences in cusp position were due to differences in the cusp shape (CS) score only. All M1 and M2 *D. bicornis* teeth had ‘high’ occlusal relief (OR) in CM(R)-S (Table 3). By contrast, both EM-S and EM(R)-S, detected differences in CS and OR. OR scores for EM-S were between ‘high’ and ‘low’, whereas the OR adjustments of EM(R)-S were between ‘high-high’ and ‘low’. CS scores for the M1 and M2 were between ‘round-sharp’ and ‘round’. Similar results were also obtained for *R. sondaicus* (Table S3), but scores were more attrition-dominated than *D. bicornis.*

**Table 3:** Cusp shape and occlusal relief score comparison between methods.

				Cusp shape		Occlusal relief		
Species	Tooth and cusp position		n	CM(R)-S	EM-S and EM(R)-S	CM(R)-S	EM-S	EM(R)-S
				0–2	0–4	0–1	0–3	0–4
*Diceros bicornis*	M1	A	21	1.1±0.3	2.0±0.6	0.0±0.0	2.4±0.5	2.1±0.6
		P		1.0±0.4	1.5±0.6	0.0±0.0	2.6±0.5	1.2±0.8
	M2	A	22	0.5±0.5	2.0±0.5	0.0±0.0	1.9±0.4	2.2±0.5
		P		0.5±0.5	1.4±0.7	0.0±0.0	1.5±0.5	0.5±0.5
*Rhinoceros unicornis*	M1	A	11	1.3±0.6	2.3±0.6	0.2±0.4	2.7±0.5	2.6±0.5
		P		0.9±0.5	2.2±0.9	0.1±0.3	2.1±0.3	2.5±0.5
	M2	A	11	1.2±0.6	2.0±0.8	0.1±0.3	2.5±0.5	2.1±0.3
		P		0.8±0.4	1.7±0.6	0.0±0.0	1.7±0.5	0.9±0.7
*Ceratotherium simum*	M1	A	7	2.0±0.0	3.9±0.4	0.9±0.4	3.0±0.0	3.0±0.6
		P		1.9±0.3	3.4±1.0	0.9±0.3	2.9±0.3	3.6±0.5
	M2	A	9	1.7±0.5	3.8±0.7	1.0±0.0	3.0±0.0	3.0±0.5
		P		1.9±0.3	3.8±0.7	0.9±0.3	2.9±0.3	3.3±0.7
*Rhinoceros sondaicus*	M1	A	6	1.0±0.0	2.0±0.0	0.0±0.0	2.0±0.0	1.5±0.5
		P		0.8±0.4	1.5±0.5	0.0±0.0	1.3±0.5	0.5±0.8
	M2	A	4	0.8±0.5	1.8±0.5	0.0±0.0	1.8±0.5	1.0±0.8
		P		0.5±0.6	0.8±0.5	0.0±0.0	1.3±0.5	0.3±0.5

The mean ±standard deviation score for cusp shape and occlusal relief for the M1 and M2 in the anterior and posterior cusp position for the classical mesowear method for rhinos of Fortelius and Solounias [6] (CM(R)-S), the extended mesowear method of Winkler and Kaiser [15] (EM-S) and the extended mesowear method adapted for rhinos (EM(R)-S). All methods use single cusp scoring. Score ranges are indicated.

In *R. unicornis*, CM(R)-S did not detect differences between cusp or tooth positions in the M1 and M2, but significant differences were detected in both cusp and tooth position by EM-S (*p*<0.05) and EM(R)-S (*p*≤0.001). The posterior M2 of *R. unicornis* had particularly high OR, relative to the other teeth, and scored ‘high’ and ‘high-high’-‘high’ (0.8±0.5) in CM(R)-S and EM(R)-S, respectively.

In *C. simum*, no significant differences were found between cusp or tooth position in the M1 or M2, with scores consistently low and blunt. The addition of the ‘flat-negative’ category resulted in more OR differences, but these differences were not significant.

### Cusp-specific Wear Signatures

**Figure 4:**
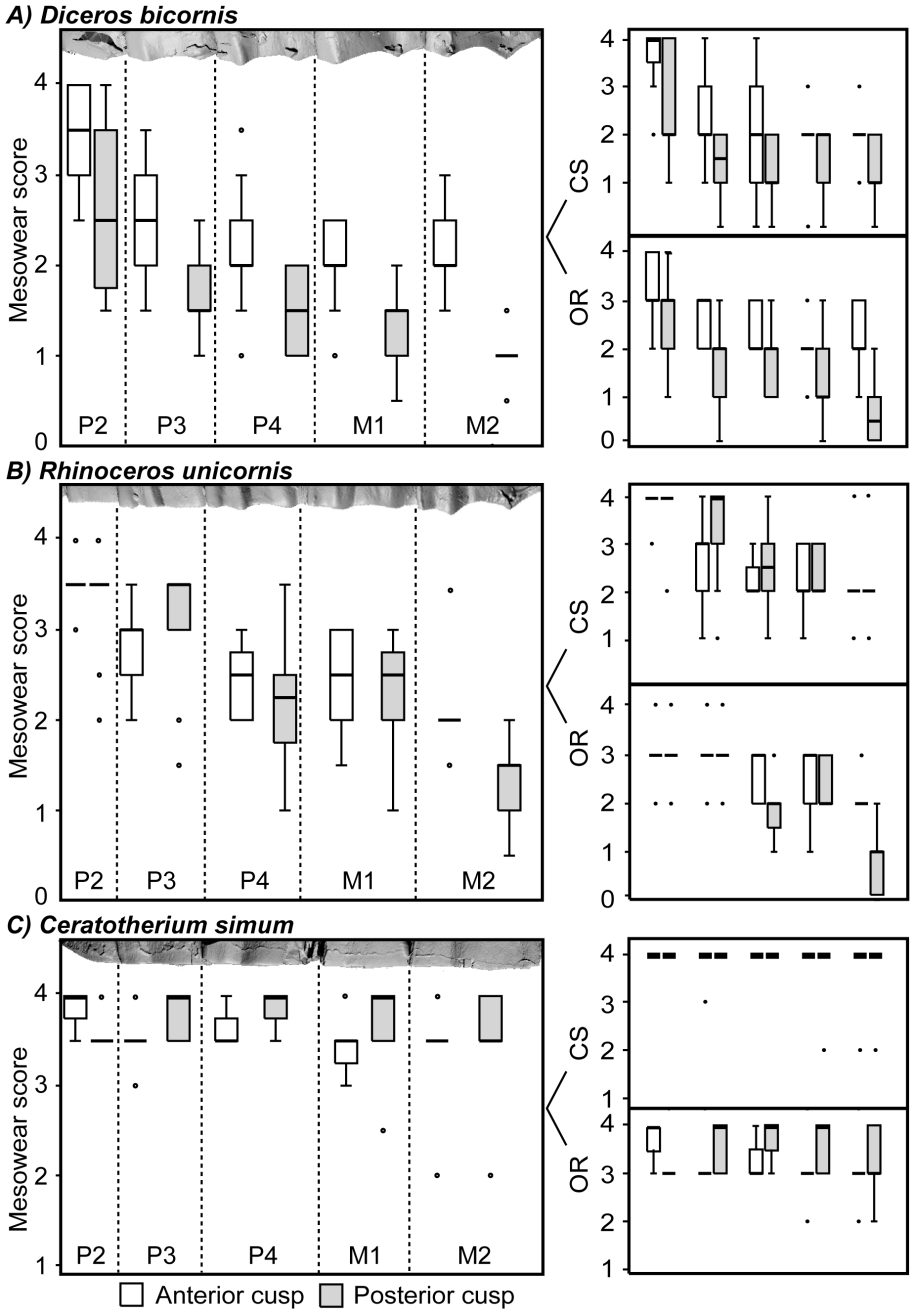
Comparison of tooth wear between and within the rhino species. Box plot of the mesowear score, cusp shape (CS) score and occlusal relief (OR) score for each the anterior and posterior cusp position of each tooth of (a) *Diceros bicornis*, (b) *Rhinoceros unicornis* and (c) *Ceratotherium simum* for the full dataset. 3D scans of maxillary tooth rows were selected based on the similarity of their mesowear scores to the sample mean. Specimen identification: NMB-1021034, AMNH-54455, NHM-752384.

When EM(R)-S was applied to the whole tooth row, in *D. bicornis* the anterior cusp was significantly blunter (*p*CS<0.05) and lower (*p*OR<0.01; *p* overall mesowear score <0.001) than the posterior cusps along the whole tooth row (Tables 4 and S3; Figure 4). In contrast, there were no significant differences in CS score between the anterior and posterior cusp position of *R. unicornis* or *C. simum*. In *R. unicornis*, the OR of the anterior M2 (2.1±0.3) was significantly lower than the posterior (0.9±0.7; *t*_15.58_ = −5.14, *p*<0.001). In *C. simum*, the anterior P2 OR was significantly lower than the posterior cusp (*t*_11.29_ = −2.45, *p = *0.032; A<P), whereas in the other tooth positions the posterior cusp was lower than the anterior cusp, with a significant difference in the P4 (*t*_14.00_ = 2.16, *p = *0.049) and no significant difference in the P3 (*t*_15.75_ = 2.00, *p = *0.063) and M1 (*t*_11.93_ = 1.92, *p = *0.070). In *R. sondaicus*, there were no differences in the overall mesowear score between the anterior and posterior cusp in the P3 (1.5 vs 1.5) or P4 (1.6 vs 1.6), but a difference between M1 (1.8 vs 1.0) and M2 (1.4 vs 0.5) caused by differences in both CS and OR.

**Table 4:** Cusp and tooth position differences in cusp shape (CS), occlusal relief (OR) and mesowear score.

		Linear mixed-effects model		Cusp differences					Tooth position differences	
Species	Score type			(Welch Two Sample t-test)					(One-way ANOVA with Dunnett’s T3 post-hoc)	
		Cusp	Tooth (Cusp)	P2	P3	P4	M1	M2	Anterior cusp	Posterior cusp
*Diceros bicornis*	CS	**<0.001**	**<0.001**	*** A>P	*** A>P	* A>P	** A>P	*** A>P	*** P2^a^>P3^b^P4^b^M1^b^M2^b^	*** P2^a^>P3^b^P4^b^M1^b^M2^b^
	OR	**<0.001**	**<0.001**	** A>P	*** A>P	*** A>P	*** A>P	*** A>P	*** P2^a^P3^ab^P4^bc^M1^c^M2^c^	*** P2^a^P3^ab^P4^b^M1^b^>M2^c^
	Mesowearscore	**<0.001**	**<0.001**	*** A>P	*** A>P	*** A>P	*** A>P	*** A>P	*** P2^a^>P3^b^P4^bc^M1^c^M2^c^	*** P2^a^>P3^b^P4^b^M1^b^>M2^c^
*Rhinoceros unicornis*	CS	0.729	**<0.001**	NS	NS	NS	NS	NS	*** P2^a^>P3^b^P4^b^M1^b^M2^b^	*** P2^a^P3^ab^P4^abc^M1^bc^M2^c^
	OR	**<0.001**	**<0.001**	NS	NS	(*) A<P	NS	*** A<P	*** P2^a^P3^a^P4^ab^M1^ab^M2^b^	*** P2^a^P3^ab^P4^bc^M1^ab^>M2^c^
	Mesowearscore	0.085	**<0.001**	NS	NS	NS	NS	** A<P	*** P2^a^>P3^b^P4^b^M1^b^M2^b^	*** P2^a^P3^ab^P4^bc^M1^b^>M2^c^
*Ceratotherium simum*	CS	0.503	0.213	NS	NS	NS	NS	NS	NS	NS
	OR	**0.013**	**0.014**	* A>P	(*)	* A<P	(*)	NS	(*)	NS
	Mesowearscore	0.22	0.081	* A>P	* A<P	* A<P	NS	NS	NS	NS

The linear mixed-effects models use the data from the extended mesowear method for rhinos (EM(R)-S) and include random effects for individual. Significance *p*-values are highlighted in **bold** for LME models and denoted as NS = not significant, (*)<0.1, * ≤0.05, ** ≤0.01 and *** ≤0.001. Dunnett’s T3 results are indicated by superscript with different letters indicating a statistical difference. Effect direction is indicated.

### Tooth-specific Wear Signatures

In *D. bicornis*, *R. unicornis* and *R. sondaicus*, a negative tooth wear gradient was exhibited along the tooth row, with wear patterns becoming less-abrasion dominated from P2-M2. In *D. bicornis*, tooth wear gradients occurred in parallel along the anterior and posterior cusp tooth row. In both *D. bicornis* and *R. unicornis,* the M2 had significantly higher OR and sharper CS than P2 in both the anterior and posterior cusp positions (Dunnett’s T3 post-hoc: *p*<0.01) (Tables 4 and S3; Figure 4). The P2 was consistently lower and blunter than the other tooth positions. Due to the cusp and tooth position differences, the posterior M2 cusp provided scores analogous with the positions used so far in the literature to infer diet. The OR of the posterior M2 cusp was significantly higher than in the other tooth positions (*p*<0.05). By contrast, there were no significant differences in OR between the anterior P4, M1 and M2 cusps. In *D. bicornis*, the CS in both the anterior and posterior cusp positions did not differ significantly between the P3 and M2 in each cusp row, respectively, but the P2 was significantly blunter (*p*<0.01). In *R. unicornis*, the anterior CS was significantly blunter in P2 than the other anterior cusp positions (*p*<0.05), and in the posterior cusp position, CS increased gradually along the tooth row. In *C. simum*, there were no significant differences in tooth position in either the anterior and posterior cusp row, with abrasion-dominated scores in every cusp and tooth position. 42% of white rhino OR scores were ‘flat-negative’, 56% ‘low’ and just 2% ‘high-low’.

## Discussion

### Mesowear Development

The results of this study indicate that extending the categories of occlusal relief (OR) and cusp shape (CS) increases the probability of detecting intra-cusp and intra-tooth wear pattern differences. Whilst all three mesowear methods evaluated were able to differentiate between the tooth wear experienced by *Diceros bicornis*, *Rhinoceros unicornis* and *Ceratotherium simum*, the extended mesowear method of Winkler and Kaiser [15] with the occlusal relief (OR) adjustments for rhinos and single cusp scoring (EM(R)-S) provided a higher level of intra-species resolution. The ‘classical’ mesowear method of Fortelius and Solounias [6] for rhinos, with single cusp scoring (CM(R)-S), provided less intra-species resolution due to the smaller number of mesowear categories. Differences in score were predominantly caused by differences in cusp shape (CS) rather than OR. CS differences were then amplified in the mesowear score due to the way the OR and CS are combined in ‘classical’ mesowear to create a score (i.e. high and sharp = 0, high and round = 1). By contrast, in EM(R)-S, significant differences were detected in OR between cusp and tooth positions, particularly in *D. bicornis.* Although the original method of Winkler and Kaiser [15] with single cusp scoring (EM-S) had higher intra-species resolution than CM(R)-S, OR scores were only exhibited in three of four categories, with no individuals attaining ‘high-high’, which reduces overall resolution in rhinos. The higher intra-species resolution provides new insights into the diets and mechanics of ingestion and mastication in rhinos.

### Limitations

Before discussing the results in more detail, some of the limitations of this study must first be stated. The sample sizes of *R. unicornis, C. simum* and, particularly, *R. sondaicus* were relatively low. Although only functional wear stages were used, ontogenetic impact cannot be excluded [11]. Due to broken cusp and wear stage exclusions, there were unequal sample sizes in each cusp and tooth position. A fully balanced subsampling approach was explored for the inferential statistics, but resulted in a substantial loss of data without a significant difference in the results or improvement to the model strength; thus samples were balanced in tooth position only. The OR boundaries were altered specifically for the family Rhinocerotidae and are not applicable to other ungulates, which limits the applications of the method. OR comparisons with other species must therefore be made with caution. However, as we used the boundary defined as ‘high’ by Winkler and Kaiser [15] as the ‘high-high’ boundary for rhinos, a simple comparison between rhinos and other ungulates is still possible when the teeth are scored with adjusted OR.

### Species-specific Wear Signatures

The OR of the posterior M2 in both *D. bicornis* and *R. sondaicus* scored ‘high’ by CM(R)-S and ‘high’ – ‘high-high’ in EM(R)-S, indicating a high relative cusp height typical of a browser. By contrast, the CS for *D. bicornis* and *R. sondaicus* were between ‘round’ and ‘round-sharp’, which is somewhat blunter than typically observed in browsers [6]. Irrespective of the scale differences, sharp cusp tips were not common within the rhino specimens studied. In both browsers, planar facets often joined at a visibly rounded cusp tip. *D. bicornis* has a two phase chewing movement, with a powerful phase I upstroke [4]. The upwards power stroke of *D. bicornis* may cause a natural rounding of cusp tips. Further research is required into the effects of chewing movement on the development of mesowear patterns.

*R. unicornis* is a mixed feeder preferring grass, consuming between 53–87% grass depending on the season [28]–[34]. In this study, mesowear scores varied, which we attribute to intraspecific variation in dietary habits. Despite the variation, OR and CS were attrition-dominated. Fortelius and Solounias [6] also report attrition-dominated wear signatures in a small sample of *R. unicornis* (n = 5), whereas Hernesniemi et al. [8] report abrasion-dominated signatures. Visually inspecting the whole tooth row, there was a subjective impression that the tooth row had lower OR than *D. bicornis* due to the flatter tooth morphology. However, the actual measurements, irrespective of OR boundaries, show that the relative height of the cusps, particularly in the posterior M2, were closer to the range of *D. bicornis* than to that of *C. simum*. These results are also concurrent with the findings of a low hypsodonty index [21] and substantial tannin-binding salivary proteins [35] in this species, which are difficult to reconcile with a grass-dominated diet. This could indicate a relatively recent evolutionary shift to a grass-dominated mixed-feeder diet or, in the case of tooth wear, indicate the consumption of a lower amount of environmental abrasives than usually associated with grass-dominated diets. Lower hypsodonty indices have been found to be associated with feeding in closed habitats, where there may be lower levels of environmental abrasives like adherent grit on grass [36]. Therefore, the higher mesowear score and lower hypsodonty index in *R. unicornis* may be due to lower intake of environmental abrasives in a riverine habitat. Furthermore, the mesowear score of browsing *R. sondaicus,* inhabiting tropical rainforests, were more attrition-dominated than browsing *D. bicornis* from drier savannah habitats, which could indicate a lower consumption of environmental abrasives by *R. sondaicus.* Analysing the acid insoluble ash (AIA) content of the faeces from *R. unicornis* and *R. sondaicus* as a proxy for the intake of endogenous dietary and exogenous environmental silica may provide more insight into the abrasiveness of rhino diets [37]. As the diets of extinct rhinos are often inferred from comparisons of tooth wear patterns of extant rhinos [6], [8], [16], understanding such patterns would offer new insights into the diets, and possibly habitats, of extinct rhinos. In addition, a mesowear method with a higher resolution could test subtler differences in wear pattern, such as habitat effects within a species across an environmental gradient.

Grazing *C. simum* had consistently low and blunt wear signatures in every tooth and cusp position, which we attribute to the highly abrasive diet. The additional OR category of ‘flat-negative’ resulted in a higher resolution of OR of P2-M2 in *C. simum* and the P2 of *D. bicornis* and *R. unicornis*, but did not significantly affect the results. In *C. simum,* 42% of OR scores were ‘flat-negative’, but, with the addition of ‘flat-negative’, the OR scores were on average higher than the CS scores (+0.0–0.9). Grazing artiodactyls generally have a higher OR than perissodactyls [6]; thus the additional category may not provide additional information for artiodactyls. However, Fortelius and Solounias [6] reported negative reliefs in equids. Thus, the flat-negative could provide more differentiation of OR in equids and should be tested in this family.

### Cusp-specific Wear Signatures

In *D. bicornis*, there were significant differences in both OR and CS between the anterior and posterior cusp within each tooth position, with the anterior cusp consistently exhibiting more abrasion-dominated wear patterns than the posterior cusp. Rhino teeth are morphologically asymmetrical, particularly in *D. bicornis*, where the anterior cusp is proportionally smaller than the posterior cusp on the ectoloph [3]. Thus, morphological differences between cusp positions may influence the mesowear score. Fortelius and Solounias [6] suggested not scoring CS affected by structural elements, such as the anterior cusp in rhinos (although evidently, the posterior cusp is also affected by its structural properties). However, OR is traditionally scored from the height of the valley between two cusps, which may result in lower OR scores relative to CS scores in asymmetrical teeth and may not provide an accurate representation of the tooth wear experienced by an individual. Although rhino teeth are particularly asymmetrical, differences in the tooth symmetry are also detected in equids [38]. In contrast to *D. bicornis*, cusp differences were less evident in *R. unicornis* and *C. simum*. Cusp differences raise questions about the adaptive relevance of tooth symmetry and also the effect of tooth symmetry on the development of mesowear patterns. One potential explanation is that, in lophodonts, asymmetrical teeth are an adaptation to heterogeneous browse (leaves and twigs). A heterogeneous occlusal surface, together with a two-phase chewing movement, may aid in browse fragmentation, whereas symmetrical teeth and a one-phase grinding chewing movement may aid in fragmentation of physically more homogeneous grass. An important question is whether the two cusps have similar or different mechanical properties – for example differences in enamel thickness or enamel structure, which might make the anterior more susceptible to abrasive wear than the posterior cusp. Such a hypothetical difference would evidently reinforce and help maintain the asymmetry of the teeth during progressing wear. Differences in cusp morphology and consistency, as well as mechanical effects of asymmetrical teeth in relation to a browse-dominated diet, represent interesting research areas for rhinoceros evolutionary ecology. Another question that arises is whether wear of the posterior cusp on an asymmetrical tooth is analogous with the wear on both cusps of a symmetrical tooth, or if there are differences. Scoring the anterior and posterior cusp separately in a single cusp score system may provide new insights into the tooth wear patterns of ungulates.

### Tooth-specific Wear Signatures

In *D. bicornis, R. sondaicus* and *R. unicornis* there was a negative tooth wear gradient along the tooth row with teeth becoming less abrasion-dominated from P2-M2. Although there were significant differences in both CS and OR along the tooth row, the influence of either measure on the overall mesowear score varied. In *D. bicornis*, OR had the most influence on the gradient, with less CS differences between P3-M2, whereas, in *R. unicornis,* CS also influenced the posterior cusp row. In addition, the cusp differences in *D. bicornis* resulted in a gradient along both the anterior and posterior tooth cusp row. Tooth wear gradients have also been reported in equids (*Equus* spp.) [17], [39] and giraffe (*Giraffa camelopardalis*) [40]. Potential explanations include several intrinsic and extrinsic factors. Tooth eruption sequence has been suggested as a potential factor contributing to tooth wear patterns [40]. However, the M1 is the first permanent tooth to erupt in rhinos, followed by the M2 and P2 [4]; thus tooth eruption sequence is unlikely to be the cause of the tooth wear gradient from P2-M2. Fortelius [4] suggests that the reason for the buccal facets dipping less steeply on anterior teeth is due to anterior teeth occluding during a later stage of the power stroke, which plunges less steeply. However, the second phase of the power stroke, a transversal movement, creates wear across the tooth, which may negate these effects. The distance from the temporomandibular joint could affect a tooth wear gradient in two different ways. Firstly, a higher bite force is exerted on posterior teeth, and, secondly, the transversal chewing movement decreases in consistency and increases in span of movement from posterior to anterior. Both factors could cause the anterior teeth to become lower and blunter as chewing becomes less consistent.

Ingesta may also influence tooth wear gradients. Schulz et al. [39] found that the grazing Grevy’s zebra (*Equus grevyi*) had decreased 3D texture fill volumes towards posterior teeth, which may be due to the unidirectional passage of food and coarse-grit contamination along the tooth row, which will affect anterior teeth more severely. In browsers, intra-species differences in tooth wear patterns may thus be more evident, because environmental abrasives accumulating on the outside of ingesta will certainly affect the anterior teeth, but will be distinctively reduced once the bolus has been mixed and surface contaminations are distributed in its volume. This in turn will reduce the average abrasive contacts to the posterior teeth occlusal surfaces. In both *D. bicornis* and *R. unicornis*, the mesowear score of the P2 was significantly more abrasion-dominated than the other tooth positions (*p<*0.05), and more abrasion-dominated than would be predicted by the wear gradient of the tooth row. Rather than resembling the attrition-dominated scores typically associated with mixed-feeding or browsing, respectively, the P2 scores were typical for grazers (anterior mean mesowear score = 3.4) and similar to *C. simum* (3.9). In *R. sondaicus*, the scores were marginally less abrasion-dominated, but still low for a browser (mesowear score = 2.5). Extant rhinos do not have incisors contributing to mastication, thus the P2, and P1 if present, are the first teeth confronted with dust and grit loaded ingesta, which may produce more wear. An alternative explanation is that the anterior teeth may have a role in food cropping, particularly in browsers, which could cause the flatter tooth wear pattern exhibited. Further research is required into the potential role of the anterior cheek teeth in the food acquisition of rhinos.

In this study, tooth wear gradients were evident in the browsers and mixed-feeders, but the grazing *C. simum* had consistently low and blunt teeth, with no gradient evident. Schulz et al. [39] also found no functional tooth wear gradients in grazing *E. grevyi*, other than texture fill volume. The consistency of mesowear patterns in *C. simum* thus indicates that the impact of abrasion does not change along the tooth row. In other words, the abrasion occurring in *C. simum* is high enough to override any other signal, including those imposed by biomechanical constraints or due to the sequence of bolus formation along the tooth row. The evident interpretation is that in grass diets, abrasive elements – whether extrinsic environmental abrasives, intrinsic dietary abrasives, or both – are much more prominent than in browse diets. Given the gradients along the tooth row observed in browsers, even within cusp positions, it appears unlikely that the absence of such a gradient in the grazer only relates to biomechanical constraints. Therefore, high or low ingesta abrasiveness provides the most parsimonious explanation of presence or absence of tooth wear gradients. A possible test of this hypothesis could be to compare mesowear between free-ranging and captive specimens, as previous studies on ruminants suggest that captive herbivores receive diets that are more homogeneous in their abrasives content, with a higher abrasiveness than typical for free-ranging browsers’ diets, but a lower abrasiveness than typical for free-ranging grazers’ diets [20], [40]. This is especially the case in black rhino, as this species is often fed with high proportion of hay in its diet in captivity.

The lack of a gradient in the grazing white rhino indicates a more uniform dental function, which is in accordance with widely observed trends in evolution resulting in increased moralization of premolars and thus increased homodonty in the post canine dentition. Although not previously recognized as an abrasion-related phenomenon, increased molarization of the premolars, particularly the P4, mostly goes together with increased hypsodonty within an evolutionary lineage. Such evolutionary trends are evident in the Equidae [41], [42], Bovidae [43], Giraffidae [44], Hyracoidea [43], some rodents [45] and are also a feature present in grazing *C. simum*. If they relate to ingesta abrasiveness, as is our hypothesis, then increased abrasiveness could drive dental functional traits into uniformity (homodonty). The phenomenon could thus highlight a general highly functional constraint, which combines both dietary adaptation and functional trade-off, when abrasion becomes a factor limiting dental and individual life expectancies.

## Conclusion

Extending the mesowear categories of occlusal relief (OR) and cusp shape (CS), and using the single cusp scoring method, facilitated a more differentiated measure of wear detecting intra-cusp and intra-tooth wear pattern differences, and provided new insights into the diets, ingestion and mastication in rhinos. Differences between species corresponded to expectations, with the exception of *R. unicornis*, whose natural diet may contain fewer abrasives than assumed by the grass-dominated diet, which may be due to a lower intake of environmental abrasives in a riverine habitat. Using the method adjustments, differential wear was detected between cusps, particularly in *Diceros bicornis* where the anterior cusps exhibited consistently more abrasion-dominated wear patterns than the posterior cusps. By contrast, cusp differences were less evident in *Rhinoceros unicornis* and *Ceratotherium simum*, with more homogeneous wear between cusps. Wear differences in cusp position may relate to morphological adaptations to specific dietary regimes. A heterogeneous occlusal surface may facilitate the comminution of heterogeneous browse, whereas a uniform, broad grinding surface may enhance the comminution of physically more homogeneous grass. In *D. bicornis, Rhinoceros unicornis* and *R. sondaicus,* there was a negative wear gradient along the tooth row, with teeth becoming less abrasion-dominated from P2-M2. There are two potential explanations. Firstly, jaw biomechanics whereby the posterior teeth experience higher bite forces and smaller and more consistent transversal movements, and secondly ingesta-specific properties, with changes to the abrasive signal from grit and dust due to bolus mixing from P2-M2. The P2 may also have a role in food cropping in *D. bicornis*. However, no gradients were observed in grazing *C. simum*; therefore high or low ingesta abrasiveness provides the most parsimonious explanation of presence or absence of tooth wear gradients. A high consumption of abrasives by grazers will override other signals, leading to a uniform wear pattern and dental function along the tooth row, which could relate to the observed evolution towards homodonty. Due to the cusp and tooth position wear differences, the posterior M2 provided scores analogous with the positions used so far in the literature to infer diet. The results of this study raise questions about the influence of tooth morphology, jaw biomechanics and ingesta-specific properties on the development of tooth wear gradients in extant and extinct ungulates, and the evolution of homodonty as a possible response to abrasion.

## Supporting Information

Table S1 **Museum and specimen information.**
(DOCX)

Table S2 **Cross-table of mesowear scores.** Mesowear scores are calculated from the mean of the occlusal relief (OR) score and cusp shape (CS) score for the extended mesowear method for rhinos (EM(R)-S). OR: hh = ‘high-high’, h = ‘high’, hl = ‘high-low’, l = ‘low’ and fn = ‘flat-negative’; CS: s = ‘sharp’, rs = ‘round-sharp’, r = ‘round’, rr = ‘round-round’ and b = ‘blunt’.
(DOCX)

Table S3 **Mesowear scores for** ***Diceros bicornis*** **,** ***Rhinoceros unicornis*** **,** ***Ceratotherium simum*** **and** ***Rhinoceros sondaicus*** **.** The mean and standard deviation of scores of cusp shape (CS), occlusal relief (CS) and the mean mesowear score for each tooth and cusp position for black, Javan, greater one-horned and white rhinos using the mesowear method developed in this study.
(DOCX)
