# Supplementary material for: Detecting Inter-Cusp and Inter-Tooth Wear Patterns in Rhinocerotids
Source: PLoS One. 2013 Dec 3;8(12):e80921. doi: 10.1371/journal.pone.0080921 (PMC3849094; doi:10.1371/journal.pone.0080921)
Supplement: Table S1 — Museum and specimen information. (DOCX) [file pone.0080921.s001.docx]

# Table S1. Museum and specimen information

| **Museum Abbreviation** | **Museum Name** | **Specimen identification** |
| --- | --- | --- |
| AMNH | American Museum of Natural History New York | 54454, 54455, 51854, 146718 |
| MHN | Museum National d’Histoire Naturelle Paris | A2277 |
| NHB | Naturhistorisches Museum Bern | 1021034 |
| NHM | Natural History Museum London | 1874.11.2.2, 1876.2.15.5, 19.7.15.511, 1907.2.26.1, 1948.1.28.6, 1962.7.6.1, 1962.7.6.5, 1962.7.6.6, 1967.7.6.4, 1976.9.26.6, 2.11.18.7, 1951.11.30.2, 72.12.30.1, 72.739, 84.1.22.1+2, 1967.8.31.4, 25.5.23.1, 52.12.9.1, 75.2384 |
| NHS | Staatliches Museum für Naturkunde Stuttgart | 32018, 7564, 1218 |
| NMB | Naturhistorisches Museum Basel | 7351, C.1798 |
| NMW | Naturhistorisches Museum Wien | 4279, 4291 |
| NRM | Naturhistoriska Rijksmuseet Stockholm | A591324, A591596 |
| OUM | Oxford University Museum of Natural History | 7118, 3827 |
| PMJ | Phyletisches Museum Jena | 651 |
| SMF | Forschungsinstitut und Naturmuseum Senckenberg Frankfurt am Main | 22660, 699, 664 |
| ZMB | Museum für Naturkunde Berlin | 35744, 40053, 41480, 46166, 83230, 83232 |
| ZMH | Zoologisches Museum Hamburg | 35744, 40053, 41480, 46166, 83230, 83232, 2551, 2552 |
| ZMZ | Zoologisches Museum Zürich | 83226, 10806, 10927 |
| ZSSM | Zoologische Staatssammlung München | 1963/160, AM416, 1912/4199, 1912/4202 |
