# Supplementary material for: Detecting Inter-Cusp and Inter-Tooth Wear Patterns in Rhinocerotids
Source: PLoS One. 2013 Dec 3;8(12):e80921. doi: 10.1371/journal.pone.0080921 (PMC3849094; doi:10.1371/journal.pone.0080921)
Supplement: Table S2 — Cross-table of mesowear scores. Mesowear scores are calculated from the mean of the occlusal relief (OR) score and cusp shape (CS) score for the extended mesowear method for rhinos (EM(R)-S). OR: hh = ‘high-high’, h = ‘high’, hl = ‘high-low’, l = ‘low’ and fn = ‘flat-negative’; CS: s = ‘sharp’, rs = ‘round-sharp’, r = ‘round’, rr = ‘round-round’ and b = ‘blunt’. (DOCX) [file pone.0080921.s002.docx]

Table S2. Cross-table of mesowear scores. Mesowear scores are calculated from the mean of the occlusal relief (OR) score and cusp shape (CS) score for the extended mesowear method for rhinos (EM(R)-S). OR: hh = ‘high-high’, h = ‘high’, hl = ‘high-low’, l = ‘low’ and fn = ‘flat-negative’; CS: s = ‘sharp’, rs = ‘round-sharp’, r = ‘round’, rr = ‘round-round’ and b = ‘blunt’.

|  |  |  | Cusp shape | | | | |
| --- | --- | --- | --- | --- | --- | --- | --- |
|  |  |  | s | rs | r | rr | b |
|  |  |  | 0 | 1 | 2 | 3 | 4 |
|  | fn | 4 | 2 | 2.5 | 3 | 3.5 | 4 |
|  | l | 3 | 1.5 | 2 | 2.5 | 3 | 3.5 |
| Occlusal relief | hl | 2 | 1 | 1.5 | 2 | 2.5 | 3 |
|  | h | 1 | 0.5 | 1 | 1.5 | 2 | 2.5 |
|  | hh | 0 | 0 | 0.5 | 1 | 1.5 | 2 |
