# Supplementary material for: Detecting Inter-Cusp and Inter-Tooth Wear Patterns in Rhinocerotids
Source: PLoS One. 2013 Dec 3;8(12):e80921. doi: 10.1371/journal.pone.0080921 (PMC3849094; doi:10.1371/journal.pone.0080921)
Supplement: Table S3 — Mesowear scores for Diceros bicornis , Rhinoceros unicornis , Ceratotherium simum and Rhinoceros sondaicus . The mean and standard deviation of scores of cusp shape (CS), occlusal relief (CS) and the mean mesowear score for each tooth and cusp position for black, Javan, greater one-horned and white rhinos using the mesowear method developed in this study. (DOCX) [file pone.0080921.s003.docx]

Table S3. Mesowear scores for *Diceros bicornis*, *Rhinoceros unicornis*, *Ceratotherium simum* and *Rhinoceros sondaicus*. The mean and standard deviation of scores of cusp shape (CS), occlusal relief (CS) and the mean mesowear score for each tooth and cusp position for black, Javan, greater one-horned and white rhinos using the mesowear method developed in this study.

| Species | | P2 | | P3 | | P4 | | M1 | | M2 | |
| --- | --- | --- | --- | --- | --- | --- | --- | --- | --- | --- | --- |
|  |  | A | P | A | P | A | P | A | P | A | P |
| *Diceros bicornis* | n | 20 | | 18 | | 23 | | 21 | | 22 | |
|  | CS score | 3.7 ±0.7 | 2.6 ±1.1 | 2.5 ±0.9 | 1.3 ±0.8 | 2.0 ±1.1 | 1.4 ±0.6 | 2.0 ±0.6 | 1.5 ±0.6 | 2.0 ±0.5 | 1.4 ±0.7 |
|  | OR score | 3.2 ±0.6 | 2.6 ±0.8 | 2.7 ±0.5 | 1.8 ±0.8 | 2.4 ±0.5 | 1.7 ±0.6 | 2.1 ±0.6 | 1.2 ±0.8 | 2.2 ±0.5 | 0.5 ±0.5 |
|  | Mesowear score | 3.4 ±0.5 | 2.6 ±0.8 | 2.6 ±0.6 | 1.6 ±0.4 | 2.2 ±0.6 | 1.5 ±0.4 | 2.1 ±0.4 | 1.4 ±0.5 | 2.1 ±0.4 | 1.0 ±0.4 |
| *Rhinoceros unicornis* | n | 9 | | 9 | | 7 | | 11 | | 11 | |
|  | CS score | 3.9 ±0.3 | 3.6 ±0.9 | 2.6 ±0.9 | 3.2 ±1.1 | 2.3 ±0.5 | 2.6 ±1.0 | 2.3 ±0.6 | 2.2 ±0.9 | 2.0 ±0.8 | 1.7 ±0.6 |
|  | OR score | 3.0 ±0.5 | 3.1 ±0.6 | 2.9 ±0.3 | 2.8 ±0.4 | 2.6 ±0.5 | 1.9 ±0.7 | 2.6 ±0.5 | 2.5 ±0.5 | 2.1 ±0.3 | 0.9 ±0.7 |
|  | Mesowear score | 3.4 ±0.3 | 3.3 ±0.7 | 2.7 ±0.5 | 3.0 ±0.8 | 2.4 ±0.4 | 2.2 ±0.8 | 2.5 ±0.5 | 2.3 ±0.6 | 2.0 ±0.5 | 1.3 ±0.6 |
| *Ceratotherium simum* | n | 7 | | 9 | | 8 | | 7 | | 9 | |
|  | CS score | 4.0 ±0.0 | 4.0 ±0.0 | 3.9 ±0.3 | 4.0 ±0.0 | 4.0 ±0.0 | 4.0 ±0.0 | 3.9 ±0.4 | 3.4 ±1.0 | 3.8 ±0.7 | 3.8 ±0.7 |
|  | OR score | 3.7 ±0.5 | 3.1 ±0.4 | 3.2 ±0.4 | 3.7 ±0.5 | 3.3 ±0.5 | 3.8 ±0.5 | 3.0 ±0.6 | 3.6 ±0.5 | 3.0 ±0.5 | 3.3 ±0.7 |
|  | Mesowear score | 3.9 ±0.2 | 3.6 ±0.2 | 3.6 ±0.3 | 3.8 ±0.3 | 3.6 ±0.2 | 3.9 ±0.2 | 3.4 ±0.3 | 3.5 ±0.7 | 3.4 ±0.5 | 3.6 ±0.6 |
| *Rhinoceros sondaicus* | n | 6 | 5 | 5 | 6 | 6 | 4 | 6 | 6 | 4 | 4 |
|  | CS score | 2.2 ±1.5 | 2.6 ±0.9 | 1.8 ±0.4 | 1.8 ±0.4 | 1.8 ±0.4 | 1.5 ±0.6 | 2.0 ±0.0 | 1.5 ±0.5 | 1.8 ±0.5 | 0.8 ±0.5 |
|  | OR score | 2.8 ±0.4 | 1.8 ±1.3 | 1.2 ±1.1 | 1.2 ±1.3 | 1.3 ±0.8 | 1.8 ±1.3 | 1.5 ±0.5 | 0.5 ±0.8 | 1.0 ±0.8 | 0.3 ±0.5 |
|  | Mesowear score | 2.5 ±0.8 | 2.2 ±1.0 | 1.5 ±0.6 | 1.5 ±0.8 | 1.6 ±0.4 | 1.6 ±0.9 | 1.8 ±0.3 | 1.0 ±0.5 | 1.4 ±0.5 | 0.5 ±0.4 |
